# Naso‐pharyngeal discharge: The first symptom of COVID‐19 infection: Report two cases from Iran

**DOI:** 10.1002/ccr3.3214

**Published:** 2020-08-06

**Authors:** Fatemeh Taghizadeh, Hassan Taghizadeh

**Affiliations:** ^1^ Student Researches Committee Psychiatry and Behavioral Science Research Center Addiction Institute Zare Hospital Mazandaran University of Medical Sciences Sari Iran; ^2^ Anesteology Ward Shahid Beheshti University of Medical Sciences Tehran Iran

**Keywords:** clinical symptom, COVID‐19, naso‐pharyngeal discharge: sinusitis

## Abstract

Naso‐pharyngeal discharge as the first symptom of COVID‐19 infection is presented in two cases. Actually, based on the presented cases here we concluded that the early clinical symptoms of COVID‐19 may be mimicked the common cold features. Appropriate diagnosis and isolation of the patients help reduce further transmission.

## Graphical abstract

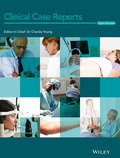

## 1 INTRODUCTION

According to the World Health Organization (WHO), coronavirus disease 2019 (COVID‐19) as a public health emergency is an international concern.^1^ First identified in Wuhan, China, it can spread rapidly with a wide spectrum of severity.^2^ There are various clinical features in COVID‐19, ranging from asymptomatic state to multi‐organ dysfunction and acute respiratory distress syndrome.^3^ The typical symptoms include cough, fever, headache, sore throat, myalgia, fatigue, breathlessness, and conjunctivitis in some cases.^4^ Thus, it is hardly distinguishable from other respiratory infections. By the end of the first week, COVID‐19 can lead to pneumonia, respiratory failure, and death.^5^ About 66.6% of the patients had cough, but only 44.7% of whom had a fever.^4^ Also, the sputum production was observed in one‐third of patients and sore throat was found in 14.0%. Less than 5% of patients had gastrointestinal symptoms, such as nausea, diarrhea, and vomiting.^4^ Fever and cough were the most common symptoms among patients with pneumonia caused by COVID‐19.^6^ In one of the first reports on the disease, Huang et al^7^ showed that the patients had dry cough, fever, dyspnea, and malaise. A suspect case is defined as a person with symptoms, including cough, sore throat, and fever, who had travelled to China or other areas of local persistent transmission and had physical contacts with confirmed COVID‐19‐infected patients.^6^ The cases may be asymptomatic or even without fever.^8^ A confirmed case is a suspect case with a positive molecular test.^8^ Nonetheless, we visited two cases in Modarres hospital clinic with naso‐pharyngeal sputum who had no history of sinusitis as the first symptom.

We presented two patients infected with COVID‐19 were followed with reverse transcription‐polymerase chain reaction (RT‐PCR) testing of throat swabs from Tehran, Iran.

## 2 CASE 1

The first case was a 45‐year‐old man who had a naso‐pharyngeal discharge for the past 6 days with no history of sinusitis. When he was visited at the clinic, he had started to have a cough, myalgia, malaise, fatigue, fever and sweating, and a positive RT‐PCR result for COVID‐19. The most important laboratory findings were included high erythrocyte sedimentation rate (ESR) and lymphopenia. Computed tomography (CT) of the chest did not show a lung involvement.

He was quarantined for 22 days at home and treated with levofloxacin, co‐amoxiclavs and acetaminophen‐codeine. Myalgia, fever, and sweating decreased after 3 days, while other symptoms continued after 28 days from onset of naso‐pharyngeal discharge. He had no dyspnea.

## 3 CASE 2

The second case was a 48‐year‐old woman with a naso‐pharyngeal discharge without a history of sinusitis. After 2 days from onset of naso‐pharyngeal discharge, she was visited at the clinic with chills, sweating, and burning in the throat. She had no fever, cough, and dyspnea and tested positive RT‐PCR. The laboratory results showed abnormal platelet count test (PLT) and LDH, lymphopenia, and high ESR. CT of the chest did not show a lung involvement.

She was quarantined for 15 days with azithromycin, cefixime, and cetirizine at home, and chills and sweating decreased, but she still felt burning in her throat after 29 days from onset of naso‐pharyngeal discharge.

## 4 DISCUSSION AND CONCLUSION

What makes the diagnosis of COVID‐19 is that these patients do not have a fever or cough on the initial presentation,^9^ and the symptoms of a more serious disease, such as dyspnea, were not present in these patients. Eventually, the report identifies the need to determine the full spectrum and natural history of clinical diseases, pathogenesis, and the duration of viral shedding related to the COVID‐19 infection to inform the clinical management and public health decisions. If the findings in this report are replicated by a naso‐pharyngeal discharge as an asymptomatic carrier, it will be too demanding to prevent COVID‐19 infection to help reduce further transmission. The mechanism, by which carriers could acquire and transmit the coronavirus, requires supplementary investigation. Appropriate diagnosis and isolation of the patients who may be at risk for COVID‐19 such as these cases are important. Timely social distancing, diagnosis, and management may help reduce the mortality rates of COVID‐19.

## CONFLICT OF INTEREST

None declared.

## AUTHOR CONTRIBUTIONS

FT: prepared this manuscript. HT: was the physician for these patients.
